# Do Successive Preterm Births Increase the Risk of Postpartum Depressive Symptoms?

**DOI:** 10.1155/2017/4148136

**Published:** 2017-05-11

**Authors:** Timothy O. Ihongbe, Saba W. Masho

**Affiliations:** ^1^Division of Epidemiology, Department of Family Medicine and Population Health, School of Medicine, Virginia Commonwealth University, Richmond, VA, USA; ^2^Department of Obstetrics and Gynecology, School of Medicine, Virginia Commonwealth University, Richmond, VA, USA; ^3^Institute for Women's Health, Virginia Commonwealth University, Richmond, VA, USA

## Abstract

**Background:**

Postpartum depression and preterm birth (PTB) are major problems affecting women's health. PTB has been associated with increased risk of postpartum depressive symptoms (PDS). However, it is unclear if PTB in women with a prior history of PTB is associated with an incremental risk of PDS. This study aims to determine if PTB in women with a prior history of PTB is associated with an incremental risk of PDS.

**Methods:**

Data come from the 2009–2011 national Pregnancy Risk Assessment Monitoring System. Study sample included 55,681 multiparous women with singleton live births in the index delivery. Multiple logistic regression was used to examine the association between PTB and PDS.

**Results:**

The risk of PDS was 55% higher in women with PTB in both deliveries (aRR = 1.55; 95% CI = 1.28–1.88) and 74% higher in women with PTB in the index delivery only (aRR = 1.74; 95% CI = 1.49–2.05), compared to women with term deliveries.

**Conclusions:**

Preterm birth is a risk factor for PDS. PTB in women with a prior history of PTB is not associated with an incremental risk of PDS. Routine screening for PDS should be conducted for all women and closer monitoring should be done for high risk women with PTB.

## 1. Introduction

Depression is a major medical problem affecting women's perinatal health. Approximately 10% to 15% of women experience postpartum depression after childbirth [1]. Postpartum depression has a devastating impact on the mother as well as the development of the child. Postpartum depression has been linked to a wide range of adverse consequences such as impaired mother–infant interactions, infant social and emotional functioning, and disruption of cognitive development of the infant [2]. In addition, postpartum depression affects marital and personal relationships, as well as having substantial negative impact on the family [3]. It decreases relationship satisfaction, causes lack of intimacy, and can lead to sexual issues, which can disrupt and negatively impact normal functioning of the family. Furthermore, women who suffer from postpartum depression have a twofold increased risk of experiencing future episodes of depression [4].

Poor birth outcomes such as preterm birth have been reported to increase the risk of postpartum depressive symptoms in women [5–7]. O'brien et al., in a longitudinal study on maternal depression following premature birth, reported that almost half the mothers of premature infants reported depressive symptoms during hospitalization, at discharge, and six weeks after discharge [8]. Voegtline and Stifter also reported that mothers of preterm babies had significantly higher levels of depressive symptoms within the first year after childbirth compared to mothers of children born full-term [9].

The increased risk of postpartum depressive symptoms observed in women with preterm birth may be due to stress [10–12]. Mothers of infants with preterm birth may experience increased stress related to feelings of helplessness, exclusion, and alienation and lack of sufficient knowledge regarding parenting and interacting with their infants [13]. Additionally, prolonged hospitalization and infant complications associated with preterm birth may aggravate the mother's feelings of helplessness and further increase her level of stress [13]. The increased level of stress may thus impact a woman's ability to adjust or transition to motherhood, increasing her likelihood of experiencing postpartum depressive symptoms [14]. Furthermore, infants born premature are more likely than full-term infants to have a more difficult temperament [15] and negative affect [5]. Such difficult infant temperament has been shown to be associated with postpartum depressive symptoms in the mother [16].

Previous preterm birth has been reported as one of the strongest determinants of a subsequent delivery of a preterm baby in women [17, 18]. However, it is unclear whether women with preterm births in the penultimate and index deliveries have an elevated risk of experiencing postpartum depressive symptoms relative to women with preterm birth in the index or penultimate delivery only. Though professional health organizations such as the American College of Obstetricians and Gynecologists (ACOG) [19], American Academy of Pediatrics (AAP) [20], American Psychiatric Association (APA), American Academy of Family Physicians (AAFP), and American College of Nurse Midwives (ACNM) recommend screening women at least once during the perinatal period for depression, over 50% of postpartum depression cases remain undetected [21]. A study by Psaros et al. reported that although about 61% of health care providers endorsed routine screening for postpartum depression, only 17% reported using a screening instrument [22]. One of the reasons for not screening is the lack of resources to implement universal screening and management of postpartum depression [23]. However, health care providers may be more apt to screen and provide targeted care to women with high risk conditions [24], such as a previous preterm birth. Understanding the interrelationship between successive preterm birth and postpartum depressive symptoms will enable health care providers to screen and manage this population of women more efficiently. This study, therefore, aims to examine if the delivery of a preterm baby in both the penultimate and index pregnancies increases the risk of women experiencing postpartum depressive symptoms above the risk observed in women with preterm birth in the penultimate or index delivery alone. We hypothesize that preterm birth in both the penultimate and index pregnancies will increase the risk of postpartum depressive symptoms.

## 2. Materials and Methods

This study utilized data from the 2009–2011 national Pregnancy Risk Assessment Monitoring System (PRAMS) and linked birth certificate data. PRAMS is a self-reported survey conducted by the Centers for Disease Control and Prevention (CDC) and state health departments. It is administered to a sample of recently delivered mothers within 2 to 6 months after delivery of a live infant and collects information on maternal behaviors, attitudes, and experiences before, during, and immediately after pregnancy. A complex multistage sampling design was utilized and data were weighted to account for sampling design, nonresponse, and noncoverage. Data were collected using mailed questionnaires and nonrespondents were contacted by telephone. Additionally, the PRAMS survey data were linked to birth certificate data to capture birth outcome and pregnancy history data. More details on the PRAMS methodology have been previously published [25] and are also available from the PRAMS website (https://www.cdc.gov/prams). The current analysis utilized deidentifiable public-use data and was approved as exempt by the Virginia Commonwealth University's Institutional Review Board.

The study sample for this analysis included multiparous women (i.e., more than one live birth) with singleton live birth in the index delivery. Women with multifetal births (e.g., twins, triplets) or whom multifetal birth status could not be ascertained and primiparous women were excluded from the study. Women with multifetal birth were excluded from the study because multifetal birth is known to increase the risk of postpartum depressive symptoms [26]. This yielded a total of 58,224 women who were eligible for the study. Of these eligible women, those without a valid record on preterm birth in the penultimate delivery (*n* = 1,728; 2.9%) and those who did not provide valid responses to questions on postpartum depressive symptoms in the current postpartum period (*n* = 815; 1.4%) were excluded from the analysis. This yielded a total sample size of 55,681 women.

The dependent variable, postpartum depressive symptoms, was measured using three items on the PRAMS questionnaire (Cronbach's *α* = 0.82) based on an algorithm developed by Ohara et al. [27]. Women were asked: (1) “Since your new baby was born, how often have you felt down, depressed, or sad?,” (2) “Since your new baby was born, how often have you felt hopeless?,” and (3) “Since your new baby was born, how often have you felt slowed down?” Each item was measured on a scale of 1: Never to 5: Always. Scores for the three items were summed to create the postpartum depressive symptoms score. Postpartum depressive symptoms score ranged from a minimum score of 3 to a maximum score of 15. Women with scores >9 were considered to have postpartum depressive symptoms. This algorithm has been shown to have a higher specificity and positive predictive value than the Edinburgh Postnatal Depression Scale, Beck Depression Inventory, and the General Depression Scale of the Inventory of Depression and Anxiety Symptoms, when they were compared to a structured clinical interview for the Diagnostic and Statistical Manual of Mental Disorders, Fourth Edition [27]. This algorithm has also been shown to closely estimate the true prevalence of postpartum depression [27].

The independent variable, preterm birth, defined as the birth of a baby born before 37 completed weeks of gestation, was created as a mutually exclusive variable consisting of four dyadic categories based on a woman's history of preterm birth in the penultimate delivery and the gestational age at birth of the index delivery. Categories include (1) successive preterm births (preterm births in both the penultimate and index deliveries), (2) preterm birth in penultimate delivery but no preterm birth in index delivery, (3) no preterm birth in penultimate delivery but preterm birth in index delivery, and (4) no preterm birth in both penultimate and index deliveries (i.e., term births). Preterm birth in the penultimate delivery was measured using an item on the PRAMS questionnaire which asked mothers “Was the baby just before your new one born more than 3 weeks before his or her due date?” and preterm birth in the index delivery was determined using the clinical estimate of gestation at birth documented on the birth certificate. Further, preterm birth in the index delivery was categorized into very preterm (<32 weeks) and moderate-to-late (32–<37 weeks) preterm birth.

Covariates including sociodemographic factors, risky behaviors, and medical and obstetric factors were examined as potential confounders. Sociodemographic factors such as age (≤24, 25–34, and ≥35 years), education (high school or less and college or higher), income (<$20,000, $20,000–49,999, and ≥$50,000), and prenatal insurance (private, Medicaid, multiple, other, and no coverage) were assessed. Race/ethnicity was categorized as non-Hispanic (NH) White, NH Black, Hispanic, and NH other. Non-Hispanic other consisted of other Asian, American Indian, Chinese, Japanese, Filipino, Hawaiian, other non-White, Alaska Native, and mixed race. Risky behaviors such as alcohol drinking and cigarette smoking were assessed using a woman's use in the last two years prior to the index pregnancy and categorized as dichotomous variables (yes or no). Medical morbidities (gestational diabetes, prepregnancy diabetes, and hypertensive disorders of pregnancy) in the index pregnancy (yes or no) were also assessed. Pregnancy intention (intended or unintended) and intimate partner violence during the index pregnancy (yes or no) were measured as dichotomous variables. Prepregnancy body mass index (BMI) was calculated as weight in kilograms divided by height in meters squared (kg/m^2^) and categorized based on the World Health Organization (WHO) classification into underweight (<18.50), normal weight (18.50–24.99), overweight (25.00–29.99), and obese (≥30.00). Stressful life events were assessed using a set of 13 questions that asked women to indicate whether they had experienced stressful events in the 12 months before giving birth (e.g., separation from partner, loss of job, bills she was unable to pay, etc.) and were categorized based on the number of stressful experiences (none, 1-2, 3–5, and ≥6). Problems with breastfeeding was measured as an abrupt termination of breastfeeding in women who commenced breastfeeding of their babies. It was categorized into 3 categories—no breastfeeding problems, breastfeeding problems, and never breastfed. Previous live births (1 and ≥2) and marital status (married and not married) were obtained from the birth certificate.

Statistical analysis was conducted using SAS-callable SUDAAN (Research Triangle Institute, Research Triangle Park, North Carolina) utilizing appropriate weights to account for the complex survey design. Descriptive analyses were conducted to assess the distribution of participants' characteristics. Prevalence of postpartum depressive symptoms was calculated and factors associated with postpartum depressive symptoms were determined. Multiple logistic regression analysis was conducted to compute the risk ratios (RRs) and 95% confidence intervals (CIs) of the association between preterm birth and postpartum depressive symptoms using the average marginal prediction as described by Graubard and Korn [28]. Average marginal predictions allow comparisons of predicted outcomes (risk) between groups of people in the population, after controlling for differences in covariate distributions between the groups [29]. Because postpartum depressive symptoms are prevalent in the study sample, the risk ratio is more appropriate for this analysis [30]. Potential confounding factors whose inclusion in the regression model resulted in a change of 10% or more in the unadjusted risk ratio were retained in the final adjusted model [31]. The final model adjusted for maternal age, race/ethnicity, stressful life events, intimate partner violence, pregnancy intention, and medical morbidities in pregnancy. Interaction between preterm birth and race/ethnicity was tested based on extant literature and was not statistically significant (*p* value = 0.5217). To determine if respondents who were excluded from the analysis due to missing data (4.3%) were different than those included in the analysis, we examined the distribution of preterm birth in the index delivery (which had complete data for all respondents). Findings show that both groups were not significantly different (8.3% versus 7.8%, *p* = 0.5435). A significance level of *α* = 0.05 was used for all analyses. All results presented are based on analyses of weighted data.

## 3. Results

Sociodemographic characteristics of the study population are shown in Table 1. Over half of the study sample were between the ages of 25 and 34 years (60.6%), NH White (56.9%), and married (66.0%) and had college education or higher (54.1%). Nearly half (46.7%) of the study sample had private insurance. Approximately 5% of women reported experiencing violence perpetrated by their partners and 42.3% reported their pregnancies as being unintended. About 10.5%, 5%, and 2.8% of the study sample reported preterm birth in the penultimate delivery only, index delivery only, and both penultimate and index deliveries, respectively. Approximately, one in nine women (11.4%) in the study population reported experiencing postpartum depressive symptoms.

The prevalence of postpartum depressive symptoms was higher in unmarried women, women who experienced stressful life events, women who were victims of intimate partner violence, those who never breastfed or had problems in breastfeeding, obese women, women who engaged in risky behaviors such as smoking cigarettes and drinking alcohol, and those whose pregnancies were unintended (Table 2). The prevalence of postpartum depressive symptoms was also greater for younger women, NH Black women, women with a household income of <$20,000, and women who were on Medicaid. Similarly, postpartum depressive symptoms were positively associated with younger maternal age, NH Blacks, unmarried women, lower household income, Medicaid and multiple insurance, overweight and obesity, medical morbidity in pregnancy, intimate partner violence victimization, unintended pregnancy, stressful life events, problems with breastfeeding, cigarette smoking, and alcohol use.

Table 3 shows the association between preterm birth and postpartum depressive symptoms. The unadjusted analyses show statistically significant associations between all categories of preterm birth and postpartum depressive symptoms. Compared to women with term births in both the penultimate and index deliveries, women with preterm birth in the penultimate delivery only, index delivery only, and both penultimate and index deliveries had significantly higher risk of postpartum depressive symptoms. However, after adjusting for maternal age, race/ethnicity, stressful live events, intimate partner violence, pregnancy intention, and medical morbidities in pregnancy, the association between preterm birth in the penultimate delivery only and postpartum depressive symptoms lost its statistical significance (aRR = 1.14; 95% CI = 1.00–1.29). On the other hand, the adjusted regression analyses show that the risk of postpartum depressive symptoms was 74% higher in women who had preterm birth in the index delivery only (aRR = 1.74; 95% CI = 1.49–2.05) and 55% higher among those with preterm births in both the penultimate and index deliveries (aRR = 1.55; 95% CI = 1.28–1.88), compared to women with term births in both deliveries. However, among women with preterm births in both the penultimate and index deliveries, as well as women with preterm birth in the index delivery only, the risk of postpartum depressive symptoms was significantly elevated only when the index delivery was very preterm (i.e., <32 weeks gestation). There was no significantly elevated risk when the index delivery was moderate-to-late preterm.

## 4. Discussion

The present study shows that women with preterm birth in the index delivery only and those with preterm births in both the penultimate and index deliveries have an increased risk of experiencing postpartum depressive symptoms. These findings are consistent with those from previous studies that reported increased risk of postpartum depressive symptoms in women with preterm birth [5, 7–9]. Although, no study has previously examined the effect of successive preterm birth on the risk of postpartum depressive symptoms, Bener reported that postpartum mothers of preterm infants with a history of preterm birth had higher prevalence of psychological distress [32], which may increase their risk of developing postpartum depressive symptoms [12]. The elevated risk of postpartum depressive symptoms observed in women with preterm birth in the current study may be due to an increase in maternal level of stress associated with infant illness and complications and concerns about the infant's outcome as explained by the preterm parental distress model [10].

Further, the study shows that women with preterm births in both the penultimate and index deliveries did not have greater risk of postpartum depressive symptoms than women with preterm birth in the index delivery only. A nonsignificant association between preterm birth and postpartum depressive symptoms in women with preterm birth in the penultimate delivery only, coupled with the lower risk of postpartum depressive symptoms observed in women with preterm births in both the penultimate and index deliveries than with women with preterm birth in the index delivery only, suggests that the risk of postpartum depressive symptoms may be driven by the experience of preterm birth in the index delivery. Furthermore, the risk of postpartum depressive symptoms in the index delivery was shown to only be significantly elevated in women with very preterm birth (i.e., <32 weeks of gestation) and was not significantly elevated in women with moderate-to-late preterm birth (32–<37 weeks of gestation). This may suggest that even though preterm birth in the index delivery increases the risk of postpartum depressive symptoms, the risk may only be evident in women with very preterm birth. This may be due to greater level of stress in women with very preterm birth [33], as higher maternal stress levels have been associated with greater postpartum depressive symptoms [11, 12].

Our findings were in contrast to the study hypothesis. The absence of a greater risk of postpartum depressive symptoms in women who had preterm births in both the penultimate and index deliveries over women who had preterm birth in the index delivery only may be explained by the acquisition of parenting skills and the development of self-efficacy in women with preterm birth in the penultimate delivery. Randomized controlled trials (RCTs) have demonstrated that interventions which addressed sources of maternal stress effectively reduced the level of stress in mothers [34, 35]. Melnyk et al., in an RCT that evaluated the efficacy of an educational-behavioral intervention program in women with preterm birth, reported that mothers who were educated on the appearance and behavioral characteristics of preterm infants and parenting them reported significantly less stress in the NICU and less depression and anxiety at two months' corrected infant age and were more positive in interactions with their infants than did comparison mothers [34]. In another RCT, Matricardi et al. examined the effects of a parental intervention to reduce parents' stress levels during the hospitalization of very preterm infants in a NICU [35]. They reported that parents of preterm infants admitted in the NICU who were taught how to observe and massage their infants in an effort to enhance parental engagement reported significantly lower levels of stress related to infants' appearance/behavior and parental role alteration than those of the control group, at discharge.

In keeping with the recommendations of the ACOG [19] and other professional health organizations, routine screening for postpartum depressive symptoms in the postpartum period for all women should be performed by health care providers using standardized, validated tools. However, beyond the routine screening for postpartum depressive symptoms, high risk women with preterm birth may require closer monitoring by their physicians. Furthermore, preterm birth may provide a teachable moment to educate women and their families on postpartum depressive symptoms. The AAP recommends that women be screened for postpartum depressive symptoms at the 1-, 2-, 4-, and 6-month child visits [20]. However, some women may not present with postpartum depressive symptoms at these screening visits or may develop depressive symptoms at a later period. The knowledge and understanding of postpartum depressive symptoms obtained at the birth of a preterm baby may thus help to enhance recognition of the onset of depressive symptoms and facilitate early presentation to their primary care provider for further evaluation. Additionally, the current movement towards single-family room NICU has been reported to increase maternal involvement in the care of preterm babies and reduce maternal stress [36]. Incorporation of single-family rooms in the NICU setting may help to increase maternal self-efficacy and ultimately reduce the risk of postpartum depressive symptoms.

This study provided important evidence regarding the relationship between preterm births in the penultimate and index deliveries and postpartum depressive symptoms. Despite the cross-sectional design of the study, temporal sequence of the exposure and outcome was established and this enabled us to calculate the risk of postpartum depressive symptoms utilizing robust statistical methods. Findings of this study should, however, be viewed in the light of some limitations. First, postpartum depressive symptoms were self-reported by women in PRAMS. There is the possibility that postpartum depressive symptoms may have been underreported due to social desirability or the stigma attached to postpartum depression. Second, we were unable to measure some important factors such as prior history of depression or depression in pregnancy, social support, and quality of mother's relationship with partner, due to nonascertainment of these factors in the PRAMS survey. Third, history of previous preterm birth was self-reported and not clinically measured. There is the possibility of recall bias in women who had their previous delivery several years back. Fourth, due to limitations of the data, we could not distinguish between spontaneous and iatrogenic preterm birth, and, lastly, study findings are not nationally generalizable as not all states in the US participate in PRAMS.

## 5. Conclusions

This study showed that women with preterm births in the index delivery only and women with preterm births in both the penultimate and index deliveries have an increased risk of experiencing postpartum depressive symptoms. While preterm birth, particularly, very preterm birth, in the index delivery is associated with postpartum depressive symptoms, a prior history of preterm birth does not present an incremental risk in postpartum depressive symptoms. Routine screening for postpartum depressive symptoms should be conducted for all women using standardized, validated tools, and closer monitoring should be done for high risk women with preterm birth. Further, the birth of a preterm baby could serve as a teachable moment to educate women about preterm birth, appropriate maternal-infant interactions that can reduce the level of stress experienced by the mother, and identification of postpartum depressive symptoms. This will enable mothers of preterm babies to develop self-efficacy in the care of their babies, as well as recognize and seek immediate care for symptoms of postpartum depression.

## Figures and Tables

**Table 1:** Sample-weighted characteristics of study participants by preterm birth, Pregnancy Risk Assessment Monitoring System, 2009–2011.

Maternal characteristics	Total study population	P−P−	P+P−	P−P+	P+P+	*p*value^b^
	*N* = 55,681^a^%	*n* = 40,504^a^%	*n* = 5,523^a^%	*n* = 6,042^a^%	*n* = 3,612^a^%	
All participants	100	81.7	10.5	5.0	2.8	—
Maternal age (years)						<0.0001
≤24	21.3	20.8	24.0	21.6	24.6	
25–34	60.6	61.1	59.3	56.0	58.0	
≥35	18.1	18.1	16.7	22.4	17.4	
Maternal education						<0.0001
High school or less	45.9	44.6	52.1	49.9	55.0	
College or higher	54.1	55.4	47.9	50.1	45.0	
Race/ethnicity						<0.0001
Non-Hispanic White	56.9	58.6	49.8	48.4	47.5	
Non-Hispanic Black	13.5	12.5	15.5	19.6	23.3	
Hispanic	22.4	21.7	27.8	23.9	21.6	
Non-Hispanic other^c^	7.3	7.2	7.0	8.2	7.53	
Marital status						<0.0001
Married	66.0	67.8	59.3	59.1	51.8	
Not married	34.0	32.3	40.8	40.9	48.2	
Household income						<0.0001
<20,000	36.4	34.4	44.7	43.7	51.4	
20,000–49,999	29.0	29.5	27.4	25.5	26.4	
≥50,000	34.6	36.1	27.9	30.9	22.2	
Insurance						<0.0001
Private	46.7	48.2	39.9	44.0	33.4	
Medicaid	39.0	37.6	45.0	41.2	52.2	
Multiple	6.7	6.7	6.9	7.4	7.4	
Other	3.9	3.8	5.0	3.7	3.9	
No coverage	3.7	3.8	3.2	3.8	3.0	
Prepregnancy body mass index (kg/m^2^)						0.0002
Underweight	3.6	3.3	4.1	5.2	6.3	
Normal weight	46.9	47.6	44.1	45.0	41.3	
Overweight	25.4	25.2	26.9	25.1	25.4	
Obese	24.1	23.9	24.8	24.8	27.0	
Medical morbidity in pregnancy^d^	20.6	18.1	29.1	32.6	41.6	
Intimate partner violence	4.9	4.5	7.1	5.6	7.5	<0.0001
Pregnancy intention						<0.0001
Intended	57.7	58.5	54.6	54.8	50.0	
Unintended	42.3	41.5	45.4	45.2	50.0	
Stressful life events^e^						<0.0001
None	30.7	31.7	24.6	30.7	23.3	
1-2	41.1	41.4	40.8	37.5	41.7	
3–5	22.4	21.7	25.9	24.5	27.0	
≥6	5.8	5.3	8.6	7.4	8.1	
Previous live birth						0.0039
1	54.1	54.5	53.3	51.1	48.4	
≥2	45.9	45.5	46.7	48.9	51.6	
Problems with breastfeeding						<0.0001
Never breastfed	20.0	19.4	22.1	22.3	25.9	
Yes	34.1	33.1	37.5	39.7	39.8	
No	45.9	47.5	40.5	38.0	34.3	
Postpartum depressive symptoms	11.4	10.7	14.1	15.4	17.0	<0.0001
Cigarette smoking^f^	25.4	24.4	29.9	27.9	31.0	<0.0001
Alcohol use^f^	60.8	61.5	57.9	59.3	54.1	0.0006

P+P−, preterm birth in penultimate delivery but no preterm birth in index delivery; P−P+, no preterm birth in penultimate delivery but preterm birth in index delivery; P+P+, preterm birth in both penultimate and index deliveries; P−P−, no preterm birth in both penultimate and index deliveries. ^a^Unweighted frequency. ^b^Chi  square for preterm birth. ^c^American Indian, Chinese, Japanese, Filipino, Hawaiian, other non-White, Alaska native, other Asian. ^d^Gestational diabetes, prepregnancy diabetes, and hypertensive disorders of pregnancy. ^e^12 months before the birth of child. ^f^Last two years.

**Table 2:** Factors associated with postpartum depressive symptoms, Pregnancy Risk Assessment Monitoring System, 2009–2011.

Maternal characteristics	Prevalence of postpartum depressive symptoms (%)^a^	Unadjusted RR (95% CI)
Maternal age (years)		
≤24	15.9	1.93 (1.69–2.20)^*∗*^
25–34	10.9	1.32 (1.17–1.48)^*∗*^
≥35	8.3	Reference
Maternal education		
High school or less	13.5	1.36 (1.26–1.48)^*∗*^
College or higher	9.9	Reference
Race/ethnicity		
Non-Hispanic White	11.5	Reference
Non-Hispanic Black	13.5	1.17 (1.06–1.30)^*∗*^
Hispanic	10.8	0.95 (0.83–1.07)
Non-Hispanic Other^b^	9.7	0.85 (0.74–0.97)^*∗*^
Marital status		
Married	9.4	Reference
Not married	15.5	1.64 (1.52–1.78)^*∗*^
Household income		
<20,000	16.4	2.30 (2.08–2.54)^*∗*^
20,000–49,999	11.6	1.63 (1.46–1.83)^*∗*^
≥50,000	7.1	Reference
Insurance		
Private	8.6	Reference
Medicaid	15.1	1.77 (1.62–1.93)^*∗*^
Multiple	13.2	1.55 (1.33–1.80)^*∗*^
Other	9.0	1.05 (0.77–1.43)
No coverage	8.0	0.94 (0.68–1.29)
Prepregnancy body mass index (kg/m^2^)		
Underweight	11.0	1.06 (0.85–1.33)
Normal weight	10.3	Reference
Overweight	11.5	1.12 (1.00–1.24)^*∗*^
Obese	14.4	1.39 (1.26–1.53)^*∗*^
Medical morbidity in pregnancy^c^		
No	10.8	Reference
Yes	14.3	1.32 (1.20–1.44)^*∗*^
Intimate partner violence		
No	10.4	Reference
Yes	31.9	3.06 (2.74–3.41)^*∗*^
Pregnancy intention		
Intended	8.7	Reference
Unintended	15.4	1.77 (1.63–1.92)
Stressful life events^d^		
None	5.0	Reference
1-2	9.4	1.89 (1.64–2.17)^*∗*^
3–5	18.2	3.67 (3.21–4.20)^*∗*^
≥6	34.5	6.95 (6.02–8.04)^*∗*^
Previous live birth		
1	11.1	0.93 (0.86–1.01)
≥2	12.0	Reference
Problems with breastfeeding		
Never breastfed	14.9	1.94 (1.74–2.16)^*∗*^
Yes	13.6	1.77 (1.61–1.95)^*∗*^
No	7.7	Reference
Cigarette smoking^e^		
No	9.1	Reference
Yes	18.3	2.01 (1.86–2.18)^*∗*^
Alcohol use^e^		
No	9.6	Reference
Yes	12.7	1.33 (1.21–1.44)^*∗*^

^a^Weighted percent. ^b^American Indian, Chinese, Japanese, Filipino, Hawaiian, other non-White, Alaska native, other Asian. ^c^Gestational diabetes, prepregnancy diabetes, and hypertensive disorders of pregnancy. ^d^12 months before the birth of child. ^e^Last two years. ^*∗*^*p* < 0.05.

**Table 3:** Association between preterm birth and postpartum depressive symptoms.

Preterm birth	Postpartum depressive symptoms	
	Crude RR (95% CI)	Adjusted RR (95% CI)^a^
P+P−	1.33 (1.17–1.51)^*∗*^	1.14 (1.00–1.29)
P−P+		
Moderate-to-late preterm birth in current delivery	1.28 (1.08–1.53)^*∗*^	1.19 (0.99–1.42)
Very preterm birth in current delivery	1.96 (1.69–2.27)^*∗*^	1.74 (1.49–2.05)^*∗*^
P+P+		
Moderate-to-late preterm birth in current delivery	1.45 (1.17–1.80)^*∗*^	1.23 (1.00–1.52)
Very preterm birth in current delivery	2.02 (1.71–2.40)^*∗*^	1.55 (1.28–1.88)^*∗*^
P−P−	Ref.	Ref.

P+P−, preterm birth in penultimate delivery but no preterm birth in index delivery; P−P+, no preterm birth in penultimate delivery but preterm birth in index delivery; P+P+, preterm birth in both penultimate and index deliveries; P−P−, no preterm birth in both penultimate and index deliveries; RR, risk ratio; CI, confidence interval; ^a^adjusted for maternal age, race/ethnicity, stressful live events in current pregnancy, intimate partner violence, pregnancy intention, and medical morbidity in pregnancy (gestational diabetes, prepregnancy diabetes, and hypertensive disorders of pregnancy); ^*∗*^*p* value < 0.05.
